# Supplementary material for: Sex-Specific Outcomes of Acute Stroke in Patients with Systemic Lupus Erythematosus: A National Inpatient Sample Study
Source: J Clin Med. 2023 Jan 6;12(2):462. doi: 10.3390/jcm12020462 (PMC9865871; doi:10.3390/jcm12020462)
Supplement: Supplementary file 1 [file jcm-12-00462-s001.zip › jcm-2059300-supplementary.pdf]

SUPPLEMENTARY MATERIAL

Supplementary Tables

Supplementary Table S1. International Classification of Disease – tenth edition (ICD-10) codes used to extract admission co-morbidities and procedures.

SLE Subtype	ICD-10 Code (Diagnosis)
Drug-induced systemic lupus erythematosus	M320
Systemic lupus erythematosus, organ, or system involvement unspecified	M3210
Endocarditis in systemic lupus erythematosus	M3211
Pericarditis in systemic lupus erythematosus	M3212
Lung involvement in systemic lupus erythematosus	M3213
Glomerular disease in systemic lupus erythematosus	M3214
Tubulo-interstitial nephropathy in systemic lupus erythematosus	M3215
Other organ or system involvement in systemic lupus erythematosus	M3219
Other forms of systemic lupus erythematosus	M328
Systemic lupus erythematosus, unspecified	M329
**Comorbidities**	**ICD-10 Code (Diagnosis)**
Atrial Fibrillation	I48.x
Sepsis	A41.x; A49.9; O75.3; O03-O07; O08.0; T88.0; T80.2; A42.7; A22.7; B37.7; A26.7; A28.2; A54.8; B00.7; A32.7; A39.2-A39.4; T81.4; O85; A40.x; A21.7; A24.1; A20.7, A48.3
Dyslipidaemia	E78.0-E78.6
Dementia	F00.x; F01.x; F02.x, F03
Smoking	F17.2x
Parkinson Disease	G20.x
Transient Ischaemic Attack	G45.x
Rheumatic Heart Disease	I05.x-I09.x
Coronary Heart Disease	I20.x – I25.x
All-cause Bleeding	D69.8; D69.9; G97.x; H11.3x; H31.3x; H35.6x; H43.1x; H92.2x; I31.2; I60.x—I62.x; I85.01; I85.11; K25.xK29.x; K31.811; K62.5; K92.0-K92.2 I97.418; I97.42; I97.618-I97.621; J60.x-J62.x; N93.8; N93.9; N95.0; R04.x; R31.0; R31.9; R58; S06.4x – S06.6x
Pulmonary Embolism	126.x

Pulmonary Hypertension	127.x
Pericarditis	I30.x – I32
Infectious Endocarditis	I33.0; I33.9
Deep Venous Thrombosis	182.x
Pneumonia	J12.x – J18.x
Chronic Obstructive Pulmonary Disease	J44.x
Shock	R57.x
Previous Cerebrovascular Disease	Z86.73

ICD-10 - International Classification of Disease – tenth edition

SLE – Systemic Lupus Erythematosus

Supplementary Table S2. Baseline characteristics on admission of male and female stroke patients with and without comorbid SLE.

	**All**	**No SLE**	**SLE - Male**	**SLE - Female**	***P* value**
N (%)	1581430 (100)	1574380 (99.55)	940 (0.06)	6110 (0.39)	
Age, years, median (IQR)	71.00 (60.00-82.00)	71.00 (60.00-82.00)	61.00 (48.00-70.00)	60.00 (48.00-71.00)	<0.001
Length of stay, days, median (IQR)	3.00 (2.00-6.00)	3.00 (2.00-6.00)	4.00 (2.00-6.00)	4.00 (2.00-7.00)	<0.001
Stroke type, n (%)					
Ischaemic	1402150 (88.66)	1395790 (88.66)	860 (91.49)	5500 (90.02)	0.152
Haemorrhagic	179280 (11.34)	178590 (11.34)	80 (8.51)	610 (9.98)	0.152
Race, n (%)					
White	1052545 (66.56)	1048905 (66.62)	585 (62.23)	3055 (50.00)	<0.001
African American	273450 (17.29)	271235 (17.23)	250 (26.60)	1965 (32.16)	<0.001
Hispanic	113340 (7.17)	112695 (7.16)	40 (4.26)	605 (9.90)	<0.001
Asian	43645 (2.76)	43485 (2.76)	25 (2.66)	135 (2.21)	<0.001
Native American	6260 (0.40)	6230 (0.40)	<11‡	§	<0.001
Other	39540 (2.50)	39375 (2.50)	15 (1.60)	150 (2.45)	<0.001
Year of admission, n (%)					
2015	128075 (8.10)	127585 (8.10)	120 (12.77)	370 (6.06)	0.020
2016	525185 (33.21)	522930 (33.21)	300 (31.91)	1955 (32.00)	0.020
2017	545390 (34.49)	542820 (34.48)	330 (35.11)	2240 (36.66)	0.020
2018	382780 (24.20)	381045 (24.20)	190 (20.21)	1545 (25.29)	0.020
Elixhauser comorbidities, n (%)					
Congestive heart failure	243035 (15.37)	241995 (15.37)	140 (14.89)	900 (14.73)	0.810
Valvular disease	152000 (9.61)	151125 (9.60)	90 (9.57)	785 (12.85)	0.001
Pulmonary circulation disease	12470 (0.79)	12410 (0.79)	<11‡	§	0.363
Peripheral vascular disease	147340 (9.32)	146665 (9.32)	150 (15.96)	525 (8.59)	0.004
Paralysis	158400 (10.02)	157645 (10.01)	95 (10.11)	660 (10.80)	0.649

Other neurological disorders	13605 (0.86)	13555 (0.86)	20 (2.13)	30 (0.49)	0.060
Chronic pulmonary disease	248220 (15.70)	246845 (15.68)	175 (18.62)	1200 (19.64)	<0.001
Diabetes mellitus (without chronic complications)	283560 (17.93)	282605 (17.95)	140 (14.89)	815 (13.34)	<0.001
Diabetes mellitus (with chronic complications)	310945 (19.66)	309890 (19.68)	135 (14.36)	920 (15.06)	<0.001
Hypothyroidism	224265 (14.18)	222940 (14.16)	75 (7.98)	1250 (20.46)	<0.001
Renal failure	261820 (16.56)	260325 (16.54)	215 (22.87)	1280 (20.95)	<0.001
Liver disease	29765 (1.88)	29610 (1.88)	25 (2.66)	130 (2.13)	0.599
Peptic ulcer disease	10600 (0.67)	10560 (0.67)	<11‡	§	0.892
AIDS	3460 (0.22)	§	<11‡	<11‡	0.491
Lymphoma	7910 (0.50)	7855 (0.50)	<11‡	50 (0.82)	0.286
Metastatic cancer	28490 (1.80)	28390 (1.80)	15 (1.60)	85 (1.39)	0.542
Solid tumour without metastasis	29160 (1.84)	29065 (1.85)	<11‡	§	0.364
Coagulopathy	70360 (4.45)	69580 (4.42)	135 (14.36)	645 (10.56)	<0.001
Obesity	211175 (13.35)	210050 (13.34)	135 (14.36)	990 (16.20)	0.013
Weight loss	67345 (4.26)	67010 (4.26)	85 (9.04)	250 (4.09)	0.007
Fluid and electrolyte disorders	375565 (23.75)	373750 (23.74)	245 (26.06)	1570 (25.70)	0.222
Anaemia (chronic blood loss)	5620 (0.36)	5545 (0.35)	<11‡	§	<0.001
Anaemia (deficiency)	193410 (12.23)	191815 (12.18)	195 (20.74)	1400 (22.91)	<0.001
Alcohol abuse	74135 (4.69)	74035 (4.70)	35 (3.72)	65 (1.06)	<0.001
Drug abuse	42765 (2.70)	42510 (2.70)	45 (4.79)	210 (3.44)	0.059
Psychoses	37525 (2.37)	37265 (2.37)	25 (2.66)	235 (3.85)	0.008
Depression	177790 (11.24)	176550 (11.21)	105 (11.17)	1135 (18.58)	<0.001
Hypertension	1355140 (85.69)	1349435 (85.71)	815 (86.70)	4890 (80.03)	<0.001
Other comorbidities, n (%)					

Atrial fibrillation	392825 (24.84)	391640 (24.88)	180 (19.15)	1005 (16.45)	<0.001
Sepsis	26480 (1.67)	26360 (1.67)	15 (1.60)	105 (1.72)	0.989
Dyslipidaemia	892075 (56.41)	888955 (56.46)	460 (48.94)	2660 (43.54)	<0.001
Dementia	188170 (11.90)	187825 (11.93)	55 (5.85)	290 (4.75)	<0.001
Smoking	284450 (17.99)	283215 (17.99)	200 (21.28)	1035 (16.94)	0.310
Parkinson disease	22790 (1.44)	22715 (1.44)	15 (1.60)	60 (0.98)	0.425
Transient ischemic attack	11240 (0.71)	11185 (0.71)	<11‡	§	0.867
Rheumatic heart disease	44050 (2.79)	43780 (2.78)	45 (4.79)	225 (3.68)	0.039
Coronary heart disease	435990 (27.57)	434345 (27.59)	285 (30.32)	1360 (22.26)	<0.001
All-cause bleeding	280530 (17.74)	279360 (17.74)	145 (15.43)	1025 (16.78)	0.470
Pulmonary embolism	10430 (0.66)	10390 (0.66)	<11‡	§	0.546
Pulmonary hypertension	33410 (2.11)	33180 (2.11)	15 (1.60)	215 (3.52)	0.002
Pericarditis	165 (0.01)	165 (0.01)	<11‡	<11‡	0.931
Infectious endocarditis	3400 (0.21)	3370 (0.21)	15 (1.60)	15 (0.25)	<0.001
Deep venous thrombosis	23650 (1.50)	23545 (1.50)	<11‡	§	0.506
Pneumonia	47750 (3.02)	47465 (3.01)	40 (4.26)	245 (4.01)	0.075
Chronic obstructive pulmonary disease	177915 (11.25)	177090 (11.25)	140 (14.89)	685 (11.21)	0.282
Shock	8825 (0.56)	8775 (0.56)	<11‡	§	0.581
Previous cerebrovascular disease	241600 (15.28)	240430 (15.27)	130 (13.83)	1040 (17.02)	0.207
Outcomes, n (%)					
In-hospital mortality	87715 (5.55)	87385 (5.55)	30 (3.19)	300 (4.91)	0.233
Length of stay >4 days	568335 (35.94)	565410 (35.91)	375 (39.89)	2550 (41.73)	<0.001
Routine discharge	541095 (34.22)	538410 (34.20)	435 (46.28)	2250 (36.82)	<0.001
Other characteristics, n (%)					
Hospital bed size					
Small	245000 (15.49)	244070 (15.50)	135 (14.36)	795 (13.01)	0.043

Medium	456330 (28.86)	454395 (28.86)	230 (24.47)	1705 (27.91)	0.043
Large	880100 (55.65)	875915 (55.64)	575 (61.17)	3610 (59.08)	0.043
Location/Teaching status of hospital					
Rural	113290 (7.16)	112905 (7.17)	70 (7.45)	315 (5.16)	0.007
Urban non-teaching	347155 (21.95)	345790 (21.96)	185 (19.68)	1180 (19.31)	0.007
Urban teaching	1120985 (70.88)	1115685 (70.87)	685 (72.87)	4615 (75.53)	0.007
Region of hospital					
Northeast	307420 (19.44)	306195 (19.45)	180 (19.15)	1045 (17.10)	0.012
Midwest	381190 (24.10)	379590 (24.11)	250 (26.60)	1350 (22.09)	0.012
South	662835 (41.91)	659540 (41.89)	410 (43.62)	2885 (47.22)	0.012
West	229985 (14.54)	229055 (14.55)	100 (10.64)	830 (13.58)	0.012
All Patient Refined Diagnosis Related Group: severity of illness subclass					
Minor loss of function	122105 (7.72)	122105 (7.76)	<11‡	<11‡	<0.001
Moderate loss of function	729410 (46.12)	727005 (46.18)	295 (31.38)	2110 (34.53)	<0.001
Major loss of function	523970 (33.13)	520365 (33.05)	520 (55.32)	3085 (50.49)	<0.001
Extreme loss of function	205930 (13.02)	204890 (13.01)	125 (13.30)	915 (14.98)	<0.001
Disposition of the patient at discharge, n (%)					
Routine discharge	541095 (34.22)	538410 (34.20)	435 (46.28)	2250 (36.82)	0.013
Transfer to short-term hospital	45290 (2.86)	45095 (2.86)	35 (3.72)	160 (2.62)	0.013
Transfer to other facility: includes skilled nursing facility, intermediate care facility, another type of facility	674400 (42.64)	671750 (42.67)	320 (34.04)	2330 (38.13)	0.013
Home health care	218020 (13.79)	216880 (13.78)	110 (11.70)	1030 (16.86)	0.013
Against medical advice	14525 (0.92)	14475 (0.92)	<11‡	§	0.013
Died	87715 (5.55)	87385 (5.55)	30 (3.19)	300 (4.91)	0.013

Discharge alive, destination unknown	385 (0.02)	385 (0.02)	<11‡	<11‡	0.013

SLE – Systemic Lupus Erythematosus; IQR – Interquartile Range;

Independent-sample Kruskal Wallis Test was used to compare differences amongst the 3 groups – No SLE, SLE-Male, SLE-Female – for this variable.

Pearson's Chi-squared Test was used to compare differences amongst the 3 groups – No SLE, SLE-Male, SLE-Female – for this variable.

‡Cell sizes ≤10 were not reported to avoid patient reidentification, according to the Healthcare Cost and Utilization Project guidelines.

§Cell output suppressed as its value would allow identification of adjacent cell sizes ≤10.

Supplementary Table S3. Results of multivariable logistic regression assessing the association between co-morbid SLE and all in-hospital outcomes amongst female patients with ischaemic and haemorrhagic stroke, stratified by ethnicity, also including the P values assessing the significance of interaction terms between SLE and ethnicity.

**All stroke types (elective admissions excluded) – Female Patients**			
		**Odds Ratio (95% Confidence Interval)**	**P – value for interaction (SLE x Ethnicity)**
**In-hospital mortality**	Whites	1.28 (0.90-1.84)	0.371
	Non-Whites	0.99 (0.64-1.55)	
**Length of stay >4 days**	Whites	1.19 (0.99-1.42)	0.824
	Non-Whites	1.16 (0.97-1.37)	
**Routine discharge**	Whites	**0.78 (0.65-0.94)**	0.848
	Non-Whites	**0.80 (0.66-0.96)**	

Models adjusted for age, hospital region, location and teaching status, stroke type, Intravenous Thrombolysis/Endovascular Thrombectomy receipt, previous cerebrovascular accident and a wide range of comorbidities (congestive heart failure, valvular disease, pulmonary circulatory disease, peripheral vascular disease, paralysis, other neurological disorders, metastatic cancer, chronic pulmonary disease, diabetes with chronic complications, diabetes without chronic complications, solid tumour without metastases, hypothyroidism, renal failure, arthropathies excluding SLE, liver disease, peptic ulcer disease, acquired immune deficiency syndrome, coagulopathy, obesity, weight loss, fluid and electrolyte disorders, deficiency anaemia, chronic blood loss anaemia, alcohol abuse, drug abuse, psychosis, depression, hypertension, previous history of cancer, lymphoma, dyslipidaemia, smoking, chronic obstructive pulmonary disease, sepsis, dementia, rheumatic heart disease, Parkinson disease, coronary heart disease, all-cause bleeding, infective endocarditis, pericarditis, pulmonary embolism, transient ischaemic attack, pulmonary hypertension, deep venous thrombosis, atrial fibrillation, pneumonia, aspiration pneumonia, shock).

Statistically significant differences ($P<0.05$) highlighted in bold.
